# International variation in distribution of ASA class in patients undergoing total hip arthroplasty and its influence on mortality: data from an international consortium of arthroplasty registries

**DOI:** 10.1080/17453674.2021.1892267

**Published:** 2021-03-01

**Authors:** Alan J Silman, Christophe Combescure, Rory J Ferguson, Stephen E Graves, Elizabeth W Paxton, Chris Frampton, Ove Furnes, Anne Marie Fenstad, Gary Hooper, Anne Garland, Anneke Spekenbrink-Spooren, J Mark Wilkinson, Keijo Mäkelä, Anne Lübbeke, Ola Rolfson

**Affiliations:** a Nuffield Department of Orthopaedics Rheumatology and Musculoskeletal Sciences, University of Oxford, UK;; b Division of Clinical Epidemiology, Geneva University Hospitals, Switzerland;; c Australian Orthopaedic Association National Joint Replacement Registry, Australia;; d Surgical Outcomes and Analysis, Kaiser Permanente, San Diego, USA;; e Department of Medicine, University of Otago, Christchurch, New Zealand;; f The Norwegian Arthroplasty Register, Department of Orthopedic Surgery, Haukeland University Hospital, Bergen, Norway;; g Department of Clinical Medicine, University of Bergen, Norway;; h Orthopaedic Surgery and Musculoskeletal Medicine, University of Otago, Christchurch, New Zealand;; i Department of Orthopaedics, Visby lasarett Institute of Surgical Scienses, Uppsala Universitet, Uppsala, Sweden;; j Dutch Arthroplasty Register, ’s-Hertogenbosch, The Netherlands;; k Department of Oncology and Metabolism, University of Sheffield, Sheffield, UK;; l National Joint Registry for England, Wales, Northern Ireland, and Isle of Man, London, UK;; m Department of Orthopaedics and Traumatology, Turku University Hospital and University of Turku, Turku, Finland;; n Division of Orthopaedics and Trauma Surgery, Geneva University Hospitals, Switzerland;; o Department of Orthopaedics, Institute of Clinical Sciences Sahlgrenska Academy, University of Gothenburg, Sweden;; p The Swedish Hip Arthroplasty Register, Gothenburg, Sweden

## Abstract

Background and purpose — A challenge comparing outcomes from total hip arthroplasty between countries is variation in preoperative characteristics, particularly comorbidity. Therefore, we investigated between-country variation in comorbidity in patients based on ASA class distribution, and determined any variation of ASA class to mortality risk between countries.

Patients and methods — All arthroplasty registries collecting ASA class and mortality data in patients with elective primary THAs performed 2012–2016 were identified. Survival analyses of the influence of ASA class on 1-year mortality were performed by individual registries, followed by meta-analysis of aggregated data.

Results — 6 national registries and 1 US healthcare organization registry with 418,916 THAs were included. There was substantial variation in the proportion of ASA class III/IV, ranging from 14% in the Netherlands to 39% in Finland. Overall, 1-year mortality was 0.93% (95% CI 0.87–1.01) and increased from 0.2% in ASA class I to 8.9% in class IV. The association between ASA class and mortality measured by hazard ratios (HR) was strong in all registries even after adjustment for age and sex, which reduced them by half in all registries. Combined adjusted HRs were 2.0, 6.1, and 22 for ASA class II–IV vs. I, respectively. Associations were moderately heterogeneous across registries.

Interpretation — We observed large variation in ASA class distribution between registries, possibly explained by differences in background morbidity and/or international variation in access to surgery. The similar, strong mortality trends by ASA class between countries enhance the relevance of its use as an indicator of comorbidity in international registry studies.

In recent years, there has been a growing interest in comparing outcomes of total hip arthroplasty (THA) between arthroplasty registries, including rates of revisions and complications, and patient-reported benefits of surgery (Paxton et al. 2011, McGrory et al. 2016, Hughes et al. 2017, Springer et al. 2017, Paxton et al. 2018). Comparing aggregate-level registry data internationally allows examination of variation in practice and outcomes due to differences in implant use, populations, and healthcare system. However, these evaluations also have limitations. Registry populations may substantially differ in patients’ preoperative characteristics and therefore may not be directly comparable when assessing outcome. As an example, comorbidity is an important predictor of outcomes of THA, including perioperative mortality and severe complications (Weaver et al. 2003, Rauh and Krackow 2004), patient-reported benefits (Judge et al. 2013, Greene et al. 2015) and the need for revision surgery (Hooper et al. 2012, Prokopetz et al. 2012). It is possible that there are differences in comorbidity level between patients undergoing surgery in different countries because of differences in population health (e.g. burden of cardiovascular disease), in health systems, and in how the former may influence access to surgery. There are, however, few published data on population dissimilarities in pre-existing comorbidity (Franklin et al. 2017). Comparisons of outcome might therefore require controlling for the population differences in the statistical analysis by stratifying or adjusting for such patient characteristics. This requires a consistent approach to the definition and the measurement of a possible confounder such as comorbidity.

Comorbidity is a multi-dimensional phenomenon that reflects the overall health status of a patient. It is strongly associated with mortality in patients undergoing THA and is an ideal candidate for adjustment in registry analyses of mortality. Several methods to measure comorbidity exist. The scoring systems vary in the type and detail of information they require (Bjorgul et al. 2010, Inacio et al. 2015). The most widely collected comorbidity system by arthroplasty registries is the ASA classification system (Lübbeke et al. 2018). The simplicity of the score underpins its widespread use, although several studies have shown variability among anesthesiologists in assigning ASA score (Ranta et al. 1997, Mak et al. 2002, Riley et al. 2014, Sankar et al. 2014). Differences in ASA class distribution and its association with mortality may arise from underlying population health variation such as obesity and cardiovascular disease prevalence, and differences in access to healthcare/surgery. Finally, differences in registry populations (e.g., age and sex), independent of comorbidity, are susceptible to modification of the distribution of ASA class and its association with mortality.

Our objectives are therefore (i) to investigate the extent of variation in the distribution of ASA class in patients undergoing THA between arthroplasty registries internationally; (ii) to explore how far any variation identified is related to other routinely collected demographic data, specifically age and sex; and (iii) to investigate the consistency between ASA class and death within the first year after surgery between the registries studied. 

## Patients and methods

### Design

We conducted an analysis of aggregated data prospectively collected from participating arthroplasty registries. The distribution of elective primary THAs by ASA class was compared between registries. The influence of ASA class on 1-year mortality after elective primary THA was investigated.

### Patients and data sources

Arthroplasty registries were eligible to take part in this study if they were full members of the International Society of Arthroplasty Registries and collected data on ASA class of patients. To ensure comparability between registries, given the variable start date on which registries collected ASA class, we restricted inclusion to cases that were elective primary THAs performed within the period January 1, 2012 to December 31, 2016. THAs for which the indication was trauma or malignancy were excluded. Only the first THA in each patient was included. For included cases, the following patient characteristics were extracted: age, sex, BMI, diagnosis (primary or secondary osteoarthritis [OA]), and ASA class. Data on death from any cause within 1-year of THA was obtained.

### Statistics

Patient -level analysis was performed by the individual registries following a standardized protocol. The aggregated data from each registry was subsequently analyzed centrally.

#### Individual registries analysis

All registries described the distribution of baseline characteristics using frequencies and proportions. Registries calculated the cumulative incidence of mortality at 1 year after index THA, both overall and by ASA class, using Kaplan–Meier survival estimates with 95% confidence intervals (CIs). Patients were censored at loss to follow-up or end of 1 year follow-up. Registries then investigated the association between ASA class and risk of 1 year mortality with Cox proportional hazards models (presented as hazard ratios [HRs] with CIs) with ASA class I defined as the referent category. The proportionality of hazards was checked visually by plotting log–log of survival against time. Regression coefficients of the Cox models were reported with their variance–covariance matrix. The univariable model was accompanied by a multivariable model adjusting for age and sex. We did not anticipate a non-linear effect of age or an interaction between age and sex, but as a test of these assumptions we did repeat the adjusted analyses, to allow for these possibilities in the 3 largest registries: Sweden, the Netherlands, and Australia. Complete case analysis was used for adjusted models.

#### Aggregate analysis across registers

Kaplan–Meier tables submitted by each participating registry were combined to create a summary life table of mortality up to 1 year. For this purpose, effective numbers of at-risk patients and estimates of mortality at intervals during follow-up were collected from each registry. The conditional mortality estimates from each registry were derived and combined using the DerSimonian model with random effects (Combescure et al. 2014) The summary mortality estimates were obtained by the product-limit of the conditional mortality estimates.

The regression coefficients of the Cox models from the individual registries’ analysis, both univariate and multivariate, were combined also using the DerSimonian model with random effects for multivariate analyses (Jackson et al. 2010). With DerSimonian and Laird’s model, the logarithms of HRs are combined across studies by calculating a weighted average. The weight of a study depends on the precision of the estimated HR: the higher the precision, the higher the weight of the study. The advantage is that studies with a larger sample size tend to have a larger weight in the meta-analysis. In addition, the between-studies variability is accounted for in the calculation of the weights. The extension of DerSimonian and Laird’s model for multivariate analyses has been used to account for the correlation between the combined HRs. Cochran Q tests and I2 statistics were used to assess the heterogeneity across registries as described previously (Higgins et al. 2003). I2 values of 25%, 50%, and 75% indicate low, moderate, and high levels of heterogeneity, respectively.

Statistical analyses were performed with R 4.0.2 for Windows (R Foundation for Statistical Computing, Vienna, Austria).

The R package mvmeta (v1.0.3) (Gasparrini et al. 2012) was used to combine logarithm of hazard ratios and the R package MetaSurv (v0.3) (Combescure et al. 2014) to combine mortality curves. The 2-sided statistical threshold for significance was 0.05.

### Ethics, funding, and potential conflicts of interest

As this was a study of anonymized aggregated data, with no individual patient data passed to the researchers, there were no ethical issues and consent was not necessary. All data collection and analysis was funded by the core funding to the authors’ institutions and organizations.

The authors have no conflicts of interest to declare 

## Results

### International variation

7 registries were included in the study. 6 were national and population-based (Australia, Finland, Netherlands, New Zealand, Norway, and Sweden) and 1 was from a healthcare organization (Kaiser Permanente, USA).

The baseline demographic data varied by registry (Table 1). The proportion of women ranged from 53% in Australia to 66% in the Netherlands. Only 9% of patients were aged under 55 years in the Netherlands compared with 30% in Kaiser Permanente. The proportion of obese patients (BMI ≥ 30) ranged from 25% in the Netherlands and Sweden to 40% in Australia, Kaiser Permanente, and New Zealand. The proportion of THAs performed for primary OA ranged from 81% in Norway to 95% in Kaiser Permanente.

**Table 1.** Patient demographics by registry. Values are count (%) unless otherwise specified

Factor	Australia	Finland	Permanente	Kaiser Netherlands	New Zealand	Norway	Sweden
Age category							
	15,306 (13)	2,726 (12)	13,176 (30)	10,866 (9.2)	6,304 (16)	3,750 (13)	7,025 (11)
55–64	27,580 (24)	5,356 (24)	15,485 (35)	24,960 (21)	10,510 (27)	6,930 (23)	14,273 (22)
65–74	39,978 (35)	8,214 (37)	7,704 (18)	44,703 (38)	13,054 (34)	11,008 (37)	25,228 (38)
75–84	25,907 (23)	5,318 (24)	6,018 (14)	32,657 (28)	7,429 (19)	6,877 (23)	16,447 (25)
≥ 85	5,610 (4.9)	801 (3.6)	1,355 (3.1)	5,150 (4.4)	1,165 (3.0)	1,274 (4.3)	2,742 (4.2)
Missing	0	0	33	155	0	0	0
Age, mean (SD)	67 (12)	67 (11)	66 (11)	69 (12)	67 (11)	68 (11)	68 (11)
BMI category							
	475 (0.9)	118 (0.6)	351 (0.8)	603 (0.8)	222 (0.8)	ND	491 (0.8)
18.5–24.9	11,384 (22)	5,093 (27)	9,370 (22.3)	24,730 (31)	5,974 (21)	ND	19,931 (31)
25–29.9	19,296 (37)	7,983 (42)	15,318 (36.5)	34,449 (43)	10,748 (38)	ND	27,664 (43)
30–34.9	12,812 (25)	4,353 (23)	10,762 (25.6)	14,920 (19)	6,981 (25)	ND	12,275 (19)
35–39.9	5,350 (10)	1,379 (7.2)	4,893 (11.6)	3,906 (4.9)	3,181 (11)	ND	3,173 (4.9)
≥ 40	2,636 (5.1)	328 (1.7)	1,327 (3.2)	1,000 (1.3)	1,054 (3.7)	ND	570 (0.9)
Missing	62,428	3,161	1,750	38,883	10,302	ND	1,611
BMI, mean (SD)	29.4 (6.2)	28.2 (4.8)	29.1 (5.6)	27.4 (4.5)	29.0 (5.6)	ND	27.3 (4.4)
Sex							
Women	60,923 (53)	12,724 (57)	25,148 (57)	77,566 (66)	20,345 (53)	18,961 (64)	37,148 (57)
Missing	0	10	30	248	0	0	0
Diagnosis (primary/secondary OA)							
Primary OA	107,480 (94)	19,336 (90)	41,599 (95)	107,230 (91)	36,080 (94)	24,075 (81)	60,466 (92)
Missing	0	1,020	0	0	0	87	0

ND, no data

Substantial variation in the proportion in each ASA class was observed (Table 2). The Netherlands and Sweden had the lowest proportions of ASA class III–IV (14% and 17% respectively), while Australia, Finland, and Kaiser Permanente had twice those proportions (34%, 39%, and 35% respectively).

**Table 2.** ASA class by registry. Values are count (%)

Factor	Australia	Finland	Permanente	Kaiser Netherlands	New Zealand	Norway	Sweden	Total
ASA class I	11,092 (10)	2,773 (13)	1,390 (3.3)	23,750 (20)	5,972 (16)	4,586 (16)	15,116 (23)	64,679 (15)
ASA class II	57,616 (55)	10,515 (48)	26,634 (62)	77,245 (66)	23,039 (61)	19,319 (65)	38,483 (60)	252,851 (60)
ASA class III	33,969 (33)	8,076 (37)	14,212 (33)	16,586 (14)	8,718 (23)	5,600 (19)	10,793 (17)	97,954 (23)
ASA class IV	1,676 (1.6)	339 (1.6)	499 (1.2)	286 (0.2)	238 (0.6)	111 (0.4)	283 (0.4)	3,432 (0.8)
Any ASA class	104,353	21,703	42,735	117,867	37,967	29,616	64,675	418,916
Missing	10,028	712	1,036	624	495	223	1,040	14,158
Total	114,381	22,415	43,771	118,491	38,462	29,839	65,715	433,074

Over all registries, the percentage of patients aged under 55 years decreased with ASA class (from 27% in ASA class I to 4.1% in ASA class IV) and the percentage of patients 85 years or older increased (from 0.7% in ASA class I to 17% in ASA class IV) (Figure). Although these broad age patterns were observed in all registries, there were substantial differences between registries in the actual proportions of patients within the same ASA class. The percentage of patients aged under 55 years in ASA class I ranged from 22% (Netherlands) to 43% (Finland), and the percentage of patients over 85 years in ASA class IV ranged from 10% (Kaiser Permanente) to 20% (Australia).

**Figure:**
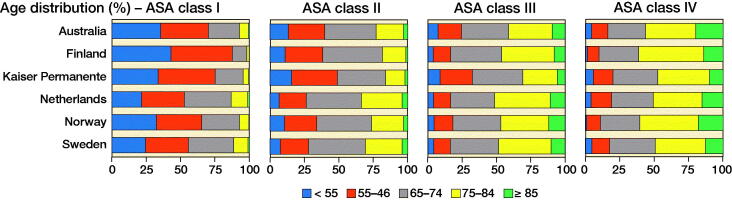
Distribution of age (years) in registries by ASA class.

### ASA and mortality within 1 year after THA

Across all registries combined, the overall mortality was 0.93% (CI 0.87–1.01). This rose with increasing ASA class, from 0.18, 0.52, 2.2, to 8.9%, respectively from class I to class IV. Although this trend was observed in all individual registries (Table 3), ASA class-specific 1-year mortality varied. Whereas mortality rates for classes I and II were almost identical between registries, there was modest variation between registries in those in classes III ranging from 1.3% to 3.1%. Variation was most extensive in class IV (range 4.5% to 16%).

**Table 3.** 1-year mortality in percentage (95% confidence interval) by registry and 1-year mortality combined across registries

			1-year mortality (%; CI)		
	ASA I	ASA II	ASA III	ASA IV	All
Australia	0.10 (0.04–0.16)	0.46 (0.40–0.52)	1.7 (1.5–1.8)	6.8 (5.6–8.0)	0.92 (0.87–0.98)
Finland	0.12 (0.00–0.26)	0.40 (0.27–0.53)	1.9 (1.6–2.2)	7.6 (4.7–11)	1.02 (0.89–1.2)
Kaiser Permanente	0.25 (0.00–0.54)	0.37 (0.29–0.45)	1.3 (1.1–1.5)	4.5 (2.6–6.5)	0.73 (0.64–0.81)
Netherlands	0.30 (0.23–0.37)	0.70 (0.64–0.76)	2.7 (2.5–3.0)	9.4 (5.9–13)	0.92 (0.87–0.98)
New Zealand	0.15 (0.05–0.25)	0.54 (0.45–0.64)	2.6 (2.3–3.0)	13 (9.0–18)	1.04 (0.94–1.1)
Norway	0.15 (0.03–0.28)	0.46 (0.35–0.56)	3.1 (2.6–3.6)	16 (8.0–23)	0.96 (0.84–1.1)
Sweden	0.16 (0.10–0.23)	0.71 (0.63–0.80)	2.5 (2.2–2.8)	8.3 (5.0–12)	0.93 (0.86–1.1)
Combined	0.18 (0.12–0.25)	0.52 (0.43–0.64)	2.2 (1.8–2.7)	8.9 (6.7–12)	0.93 (0.87–1.0)

Distribution of age (years) in registries by ASA class.

Unadjusted and age- and sex-adjusted HRs for the association between ASA class and 1-year mortality are given in Table 4. The pooled unadjusted HR confirmed an increasing 1-year mortality with increasing ASA class. This rose from an HR of 3.2 (CI 2.3–4.3) when comparing ASA class II with I, to a substantially higher HR of 59 (38–95) when comparing ASA class IV with I. There was moderate heterogeneity in the individual registries (I2 around 50%), with lowest HRs in Kaiser Permanente and the Netherlands. As an example, the unadjusted HRs in Netherlands were half those in Sweden and Finland.

**Table 4.** Meta-analysis of unadjusted and age- and sex-adjusted hazard ratios for the association between ASA classes and 1-year mortality

ASA I as reference	ASA II	Unadjusted HR (CI) ASA III	ASA IV	ASA II	Adjusted HR (CI) ASA III	ASA IV
Registries						
Australia	4.5 (2.5–8.2)	17 (9.0–30)	69 (38–129)	2.7 (1.4–4.9)	6.9 (3.8–13)	22 (12–41)
Finland	3.3 (1.0–11)	16 (5.0–50)	69 (21–229)	2.0 (0.6–6.4)	6.8 (2.1–22)	24 (7.0–84)
Kaiser Permanente	1.5 (0.5–4.7)	5.3 (1.7–17)	19 (6.0–64)	1.0 (0.3–3.2)	2.7 (0.9–8.4)	7 (2.0–25)
Netherlands	2.2 (1.7–2.8)	8.8 (6.9–11)	32 (21–50)	1.4 (1.1–1.8)	4.2 (3.2–5.4)	14 (9.0–22)
New Zealand	3.6 (1.8–7.1)	18 (9.0–34)	97 (47–204)	2.3 (1.2–4.5)	8.0 (4.0–16)	34 (16–74)
Norway	3.1 (1.3–7.1)	21 (10–48)	120 (47–309)	1.7 (0.7–3.9)	7.7 (3.3–18)	34 (13–91)
Sweden	4.4 (2.9–6.6)	16 (10–24)	54 (30–95)	3.0 (2.0–4.6)	8.6 (5.6–13)	28 (16–50)
Pooled HR	3.2 (2.3–4.3)	14 (10–19)	59 (38–93)	2.0 (1.4–2.7)	6.1 (4.4–8.5)	22 (15–32)
P-value **^a^**	< 0.001	< 0.001	< 0.001	< 0.001	< 0.001	< 0.001
Heterogeneity						
Q Cochran	11	14	14	12	13	10
Cochran test (p)	0.06	0.03	0.03	0.06	0.05	0.11
I^2^ (%)	50	57	58	51	53	42

**^a^** p-value for testing the null hypothesis that the pooled HR equals 1.

After age and sex adjustment the HRs were lowered by half: both the pooled as well as registry-level HRs. However, not all of the effect of ASA on mortality could be captured by this adjustment: the increases in the pooled HRs with increasing ASA classes were attenuated but there were still 2.0-fold, 6.1-fold, and 22-fold (CI as shown) increases in mortality in ASA classes II, III, and IV respectively compared with class I. Although all registries showed this trend, there was moderate heterogeneity across registries, and I2 statistics were around 50% with flatter rises in Kaiser Permanente and in the Netherlands compared with the other registries. As mentioned in the methods, we had assumed the effect of age would be linear. To test this assumption, we repeated the analysis in the 3 largest registries (Australia, Sweden, and Netherlands) introducing a non-linear effect of age and an age–sex interaction term. Results (not shown) did not modify sensitively the adjusted HRs for ASA classes. As these 3 registries represent around 70% of the patients included in our study we have no reason to suspect that these findings are not generalizable to the other registries. 

## Discussion

First, we have shown there was a large variation in the distribution of ASA class in patients undergoing THA. Second, given the demographic differences between registries, there were also differences in age distribution of the registry populations within ASA classes. The third conclusion relates to the association between ASA classes and 1-year mortality. Across all registries, worsening ASA class was associated with greater 1-year mortality but the magnitude of the unadjusted relationship differed between registries. Age and sex adjustment was only able to capture about half to two-thirds of the impact of ASA class on mortality, though this was consistent. After adjustment a moderate between-registry heterogeneity between ASA classes and mortality remained.

There are a number of methodological issues to consider in interpreting these findings, some of which could lead to bias in the results. The underlying aim of the study was to investigate the extent of variation in comorbidity of patients undergoing THA in different countries. Because ASA class is widely accepted as a useful guide to postoperative mortality and complications, and it is the only measure routinely collected by the registries, it was used as the proxy for comorbidity (Rauh and Krackow 2004, Hackett et al. 2015, Visser et al. 2015). Allocation of ASA class in clinical practice is subject to inter-rater variation, and as such to random error (Sankar et al. 2014). This adds noise to comparisons between populations, making it more difficult to detect true underlying differences. Despite this potential for underestimation of variation, substantial differences in ASA class were observed between the populations covered by these registries.

There are likely to be between-country differences in scoring, and specifically issues related to local rules about the availability of surgery or “upcoding”: at different times and in different jurisdictions, there may have been advantages or disadvantages for healthcare providers to under- or over-estimate the ASA class to support their service. These issues might lead to inaccuracies in ASA class allocation, the extent or direction of which is unknown.

There are other methodological issues to consider in interpreting these findings. First, there was a modest amount of missing data on ASA (< 2% overall), but with completeness at 90% or greater in the registries studied it is unlikely to be important. There were also minor differences in the number of years with available data in the different registries, but not of sufficient magnitude to be concerned about secular changes. Third, we used meta-analytic approaches, which are commonly used in clinical research to combine studies, to analyze survival data pooled across registries. The advantage of these approaches is that they can detect heterogeneity between studies. Therefore, they were appropriate to investigate the consistency of the association between ASA classes and mortality over registries.

ASA class has substantial international acceptance as a useful measure of morbidity to identify those with the greatest hazards following surgery. Thus, it is important to consider the possible explanations for the substantial differences in ASA distribution. In addition to scoring differences there are 2 other principle groups of reasons, which are (i) underlying population differences in general health covering those factors that would be reflected in the ASA class and (ii) differences in the healthcare systems that either encourage or discourage surgery in those with greater underlying health problems.

There is no published data on ASA class in the general populations in these countries, given that the tool is used only in patients selected for surgery. It is therefore relevant to consider other data to suggest that there are country differences in general health. The higher ASA classes particularly focus on cardiovascular disease (CVD). The Global Burden of Disease initiative publishes data on CVD deaths by country (Institute for Health Metrics and Evaluation 2017). The latter rate does vary between the countries covered and appears to be broadly related to the data we observed on ASA class. Thus, from the 2 countries with the highest and the lowest ASA class III proportion, Finland and the Netherlands, Finland has the highest CVD mortality and the Netherlands the lowest of the countries included.

In addition, there may be differences in healthcare coverage. The Kaiser Permanente registry is a healthcare organization registry and an exception in that the other registries are all national in their scope and during the period of the data collection > 90% complete in their population coverage. Kaiser Permanente showed the lowest proportion of ASA class I, the lowest 1-year mortality in ASA classes III and IV, and the lowest age- and sex-adjusted HRs. The main difference in the data from Kaiser Permanente is in the (lesser) effect of ASA class on mortality. This might suggest that being in ASA class III in Kaiser Permanente represents a healthier cohort than being in the same class in populations with universal healthcare coverage as Kaiser Permanente only covers the subgroup that enrolled in its healthcare plan (Wilper et al. 2009). However, the ASA distribution in KP is consistent with other US reports. A further potential concern with Kaiser Permanente is that, not being population based, attrition could be higher than in the national registers. However, Kaiser has a lower attrition rate than might be expected. Unlike most US healthcare systems, Kaiser has a 100% capture rate of patients who have an arthroplasty and a very low rate of members who leave the system, which are tracked through their membership enrolment. Over 19 years, only 8% of all the patients in KP’s registry have left the system. Other, more subtle factors can influence mortality risk, which cannot be easily captured in an analysis such as this (Woolhandler and Himmelstein 2017).

There are also a few potential confounders to consider. We specifically did not adjust for BMI, given the role of obesity in ASA assignment (Mak et al. 2002). Adjustment for BMI could have masked the effect of comorbidity that was the underlying aim of the study. There may be other unknown confounders that could have been adjusted for, such as socio-economic status. However, the relationship between socio-economic status (SES) and ASA grade is complex. There are clear associations between SES and multimorbidity (Barnett et al. 2012) and it is likely, because of this link, that, after adjustment for SES, differences between countries in ASA may be attenuated.

In conclusion, there are substantial differences in ASA class distribution between the national registries, for which the most plausible explanation is between-country differences in the underlying health status and healthcare access, as well as in scoring. The similar and strong mortality trends by ASA class between countries enhances the relevance of the use of ASA class as an indicator of comorbidity in international registry studies.
